# Palladium nanozyme loaded metal-organic framework eyedrops alleviate oxidative stress for dry eye treatment

**DOI:** 10.1186/s12951-026-04407-0

**Published:** 2026-04-10

**Authors:** Yajia Wang, Jiang Chen, Renjie Zhang, Chunyi Weng, Di Hu, Wenpei Jiang, Yingying Zhou, Quankui Lin

**Affiliations:** National Engineering Research Center of Ophthalmology and Optometry, School of Biomedical Engineering, School of Ophthalmology and Optometry, Eye Hospital, Wenzhou Medical University, Wenzhou, 325027 China

**Keywords:** Nanozyme, Dry eye, ROS, Pd-loaded MOF nanoparticle, Cell protection

## Abstract

Dry eye is a multifactorial ocular disorder caused by reduced tear secretion, poor tear quality, or excessive tear evaporation, which is a major public health issue worldwide. Oxidative stress on the ocular surface leading to excessive reactive oxygen species (ROS) production is considered a primary factor in the pathogenesis of dry eye. Herein, we designed a palladium (Pd) -loaded metal-organic framework (MOF) nanoparticle (PdM) as nanozyme eyedrops for dry eye treatment via efficient removal of various ROS. Experimental results confirmed that PdM nanozyme has multiple antioxidant enzyme activities and could scavenge a variety of free radicals. Furthermore, in vitro studies demonstrated its capacity to alleviate excess intracellular ROS, protect cytoskeletal structures and intercellular tight junctions. Treatment outcomes in animal models of dry eye confirmed its therapeutic efficacy in reducing corneal epithelial cell damage and inflammation, offering a novel approach for dry eye treatment.

**Graphical Abstract:**

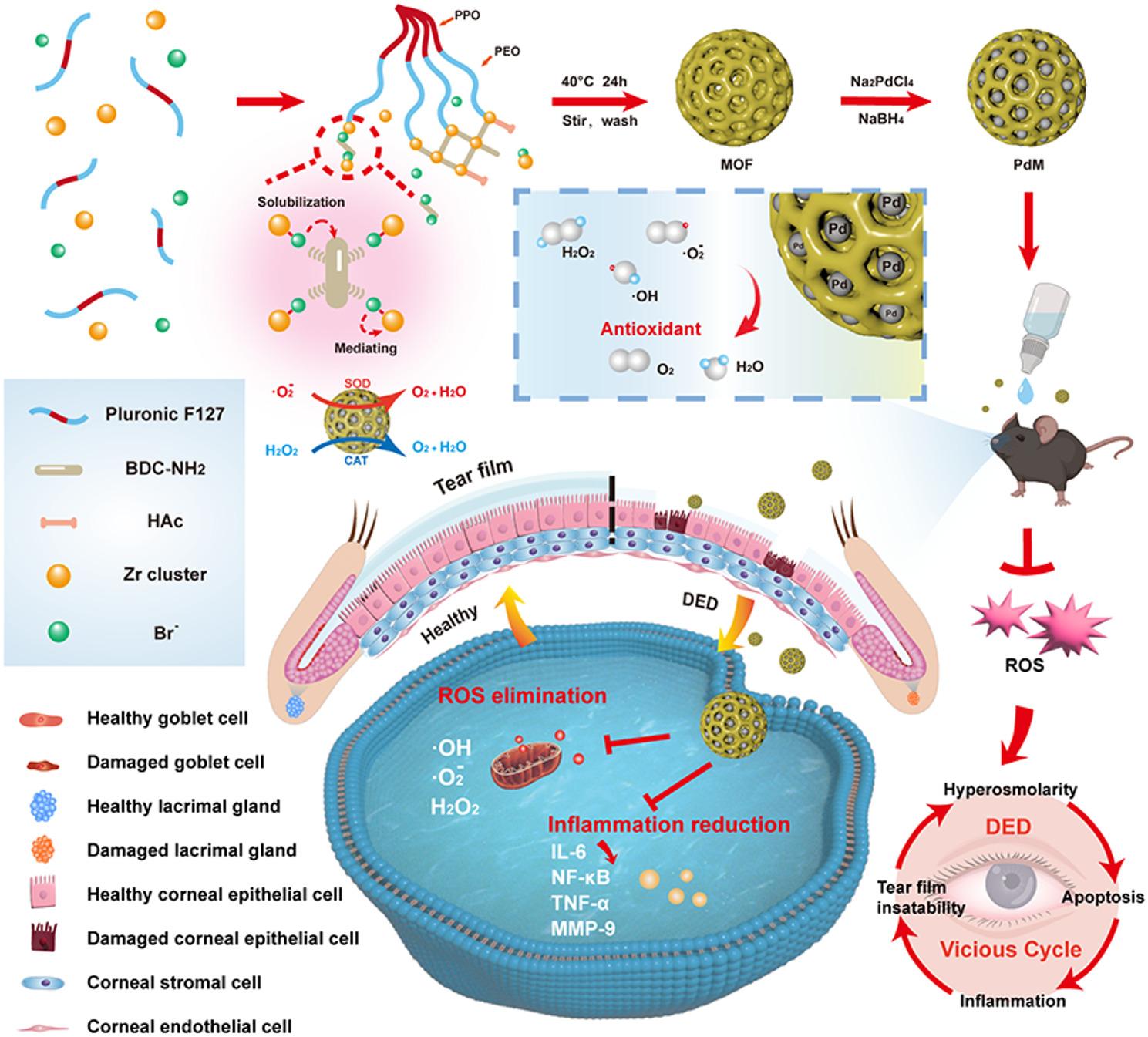

## Introduction

Dry eye syndrome (DED) is a multifactorial ocular disorder caused by reduced tear secretion, poor tear quality, or excessive tear evaporation [1, 2]. Characterized primarily by the disruption of tear film homeostasis, it presents with ocular symptoms such as dryness, tearing, foreign body sensation, and visual fatigue [3]. It is estimated that between 5% and 50% of the global population suffers from this condition, making DED a recognized global public health issue [4].Although the pathogenesis of DED remains unclear, excessive reactive oxygen species (ROS) leading to ocular surface oxidative stress and inflammation are considered key factors in its development [5, 6]. Oxidative stress arises from an imbalance between ROS levels and antioxidant defense mechanisms, potentially associated with ocular surface inflammation and hypertonic tear fluid conditions [7].

ROS are the products of aerobic metabolism within the mitochondrial respiratory chain, including superoxide anion (O₂·⁻), hydroxyl radical (·OH), hydrogen peroxide (H₂O₂), and singlet oxygen (·O₂) [8]. The relationship between excessive ROS production, lipid peroxidation-related membrane damage, protein oxidation, and inflammation has been confirmed in animal models and clinical studies [9–11]. Excessive ROS can damage proteins, lipids, and even DNA, leading to ocular surface inflammation and hypertonicity of tears. This further stimulates ROS production, creating a vicious cycle of DED [12]. Therefore, inhibiting excessive ROS generation emerges as a potential new therapeutic strategy for improving DED treatment. Antioxidants used to treat DED have demonstrated symptom-relieving capabilities in both animal models and patients, suggesting that scavenging excess ROS may offer a novel pathway for developing DED treatment regimens [13, 14].

The clinical treatment of DED currently focuses primarily on alleviating ocular surface discomfort, reducing ocular surface inflammation, and restoring the normal anatomical and physiological functions of the ocular surface and tear film [15]. Artificial tears, anti-inflammatory medications, and topical corticosteroids remain among the most commonly used treatments for DED [16]. However, these drugs only temporarily relieve symptoms by moisturizing and lubricating the ocular surface for a short period and cannot fundamentally cure DED [17]. Nanozymes are nanomaterials exhibiting enzyme-like activity [18–22]. Due to their ease of synthesis, tunable catalytic activity, high stability, and low cost, they have aroused significant interest among researchers [23–26]. Over the past decades, numerous nanomaterials have been discovered to possess mimetic activities of enzymes such as oxidase (OXD), peroxidase (POD), catalase (CAT), and superoxide dismutase (SOD), leading to extensive biomedical applications [27, 28]. Our group previously conducted several research on nanozyme treatment for DED, demonstrating its feasibility in this therapeutic context [29–31]. As a type of nanozyme, palladium nanozyme exhibits broad ROS scavenging capabilities and multiple mimetic enzyme activities [32, 33]. Their surfaces feature diverse ROS-binding sites that eliminate oxidative stress, mimicking superoxide dismutase and catalase to clear ROS and restore the balance between ROS production and antioxidant enzymes [34]. In preclinical studies, palladium nanozyme has demonstrated useful capabilities in treating neurodegenerative diseases and other conditions involving oxidative stress as radical scavengers, indicating potential intracellular ROS scavenging capacity and excellent biocompatibility [35–37].

In this study, we designed palladium (Pd) -loaded metal-organic framework (MOF) nanoparticles (PdM) nanozyme as eyedrops for DED treatment. The PdM nanozymes exhibit multiple enzyme-mimetic activities and broad ROS scavenging capabilities, enabling efficient removal of various ROS. In vitro studies not only show the good biosafety of the PdM nanozyme, but also have shown that it can effectively scavenge excessive intracellular ROS, protect cytoskeletal structure and intercellular tight junctions. The DED animal experiment results have verified that PdM nanozyme could significantly reduce ocular tissue damage and inflammatory response, providing an innovative solution for the treatment of DED.

**Scheme 1:**
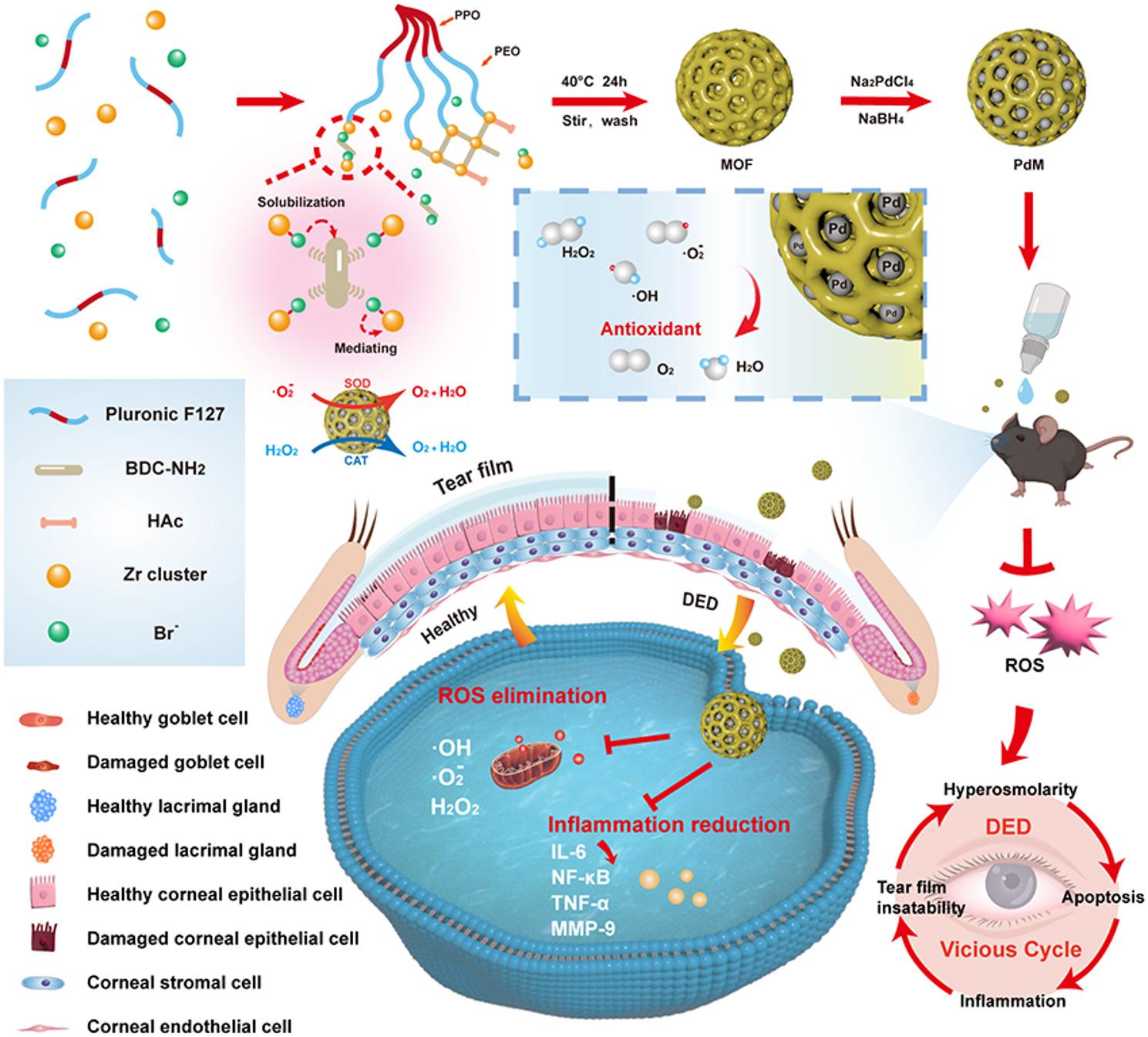
Illustrative scheme of the synthesized PdM and the therapeutic effect and mechanism of PdM for DED treatment

## Materials and methods

### Materials

Zirconium(IV) oxynitrate hydrate (ZrO(NO₃)₂·6 H₂O, 99.5%), acetic acid (AA, 99.5%), 1,3,5-trimethylbenzene (TMB, 97%), ethanol (≥ 99.8%), sodium tetrachloropalladate (II) (Na₂PdCl₄, 98%), 2,2-diphenyl-1-picrylhydrazyl (DPPH, 96%), 2,2′-Azinobis(3-ethylbenzothiazoline-6-sulfonate) (ABTS, 98%), sodium persulfate (Na_2_S_2_O_8_, 99%) and hydrogen peroxide standard solution (H₂O₂, 1000 µg/mL in H₂O) were purchased from Macklin Biochemical Co., Ltd. (China). Pluronic^®^ F-127, potassium bromide (KBr) and sodium borohydride (NaBH₄, 98%) were purchased from Sigma-Aldrich (USA). 2-aminoterephthalic acid (BDC-NH₂, 98%) was obtained from Aladdin Biochemical Technology Co., Ltd. (China). CM-H₂DCFDA (general oxidative stress indicator) was purchased from Invitrogen. DMEM/F12 (1:1) Basic (1X), 0.25% Trypsin-EDTA (1X) and 1% Penicillin-Streptomycin were purchased from Gibco (USA). Fetal bovine serum (FBS) was purchased from Lonsera (China). Scopolamine hydrobromide (Scop) was purchased from Adamas-beta. The human corneal epithelial cells (HCECs) originated from ATCC in the USA. Fluorescein sodium ophthalmic strips and a phenol red thread tear test were obtained from Tianjin Jingming New Technological Development Co. Ltd. (China). The total superoxide dismutase (SOD) assay kit with WST-8, catalase (CAT) assay kit, cell counting kit-8 (CCK-8), Calcein-AM/PI cell viability/cytotoxicity assay kit and anti-fluorescence quenching mounting medium containing DAPI were purchased from Beyotime (China).

### Cell culture

The human corneal epithelial cells (HCECs) used in this study were purchased from ATCC (USA). HCECs were cultured in DMEM/F-12 containing L-glutamine. The culture medium was supplemented with 15% fetal bovine serum, 5 µg/mL insulin, 10 ng/mL human epidermal growth factor, and 1% streptomycin/penicillin. The cells were incubated in 5% CO_2_ at 37 ^◦^C.

### Preparation of MOF

100 mg Pluronic ^®^ F-127 was dissolved in 6mL deionized water, then 50 µL TMB was added and stirred for 30 min. Then 0.8 mL AA and 240 mg KBr were added to the mixture and stirred thoroughly. 320 mg ZrO (NO_3_)_2_·6H_2_O and 100 mg BDC-NH_2_ were added and stirred at 40 ℃ for 24 h. After the completion of the reaction, the resultant MOF nanoparticles were collected by centrifugation (10,000 rpm, 10 min) and washed twice with deionized water and ethanol. Finally, the product was freeze-dried under vacuum for 48 h (Christ ALPHA 2–4 LD plus, GER).

### Preparation of PdM

20 mg of MOF was dissolved in 2 mL of ethanol and 2 mL of deionized water, then thoroughly dispersed by ultrasonication. 3.6 mg of Na_2_PdCl₄ was added to the mixture and stirred for 1 h in an ice-water bath. Then, 5 mL of a NaBH₄ (4.5 mg) aqueous solution was added to the mixture, which was stirred in an ice-water bath for 1 h. The product was then centrifuged at 10,000 rpm for 10 min to obtain a precipitate, which was washed three times with deionized water. The product was freeze-dried under vacuum (Christ ALPHA 2–4 LD plus, GER) for 48 h and store it at 4 °C in the dark. Additionally, the preparation method for PdM-FITC is as follows: 5 mg of PdM and 5 mg of FITC (Sigma) were dispersed in 1 ml of carbonate-bicarbonate buffer solution (pH = 9.4) and stirred overnight at 4 °C in the dark. After the reaction had finished, any unbound FITC was removed by dialysis using a carbonate-bicarbonate buffer solution (MWCO = 1000). The PdM-FITC solution was stored at 4 °C in the dark.

### Characterization

The morphology of the samples was observed by field emission scanning electron microscope (SEM, Regulus SU8100, Hitachi, Japan) and field emission transmission electron microscope (TEM, FEI Tecnai G2 F30, FEI, USA). Dynamic light scattering (DLS, Zetasizer Lab, Malvern, UK) was used to evaluate the hydrodynamic diameter, polymer dispersion index (PDI), and zeta potential of the samples. The crystal structure of the samples was characterized using X-ray diffraction (XRD, Ultima IV, Rigaku, Japan). The elemental composition and valence state distribution of the samples were evaluated using X-ray photoelectron spectroscopy (XPS, EscaLab 250Xi, Thermo Scientific, USA). Thermogravimetric analysis (TGA) of the samples was performed using a thermogravimetric analyzer (NETZSCH, STA 449 F3, Germany) to analyze the thermal stability and elemental composition. The optical density (OD) values of the samples were measured using a SpectraMax 190 microplate reader (Molecular Devices, USA).

### Enzyme-like activity and ROS scavenging capacity of PdM

#### SOD-like and CAT-like enzyme activity

Total Superoxide Dismutase (SOD) Assay Kit with WST-8 and catalase (CAT) assay kit were used to validate the SOD and CAT enzyme-mimetic activity of the samples. Following the instructions for the SOD assay kit, the following reagents were sequentially added to the 96-well plate: 20 µL of PdM at different concentrations (25, 50, 100, and 250 µg/mL), 20 µL of enzyme working solution, 160 µL of WST-8/enzyme working solution, and 20 µL of reaction initiation working solution. After incubating at 37 °C for 30 min, the optical density (OD) values of the samples were measured at 450 nm using a microplate reader (M5 SpectraMax, Thermo Fisher Scientific, USA). After the CAT reacts with an abundance of hydrogen peroxide (H₂O₂), any residual H₂O₂ can be oxidized to form a red substrate. Following the instructions for the CAT assay kit, 20 µL of PdM at different concentrations (25, 50, 100 and 250 µg/mL), 20 µL of catalase assay buffer and 10 µL of 250 mM H₂O₂ were incubated at 25 °C for 4 min. Next, 450 µL of peroxidase reaction stop solution were added and the mixture vortexed. 40 µL of peroxidase detection buffer were pre-placed in a clean centrifuge tube and 10 µL of the reaction mixture from above were introduced. Subsequently, 10 µL of the solution from the previous step were added to a 96-well plate, followed by 200 µL of color development solution. After incubation at 25 °C for 30 min, the absorbance of the samples were measured at 520 nm.

#### H_2_O_2_ scavenging activities of PdM

Since the decomposition of H₂O₂ produces water (H₂O) and oxygen (O₂), the amount of decomposed H₂O₂ can be calculated by measuring the oxygen content in the solution.

Different concentrations (25, 50, 100, and 250 µg·mL⁻¹) of PdM solutions were mixed with 40 mM H₂O₂ solution at a 1:1 volume ratio and incubated at room temperature for 10 min. An electrochemical analyzer (Leici, DZB-712 F, China) was used to measure the dissolved oxygen concentration in each group.

#### ROS-scavenging activities of PdM

ABTS^·+^ and DPPH scavenging assays were employed to validate the radical scavenging capacity of PdM. 2,2’-azino-bis(3-ethylbenzothiazoline-6-sulfonic acid) (ABTS) is a green organic anion. ABTS is oxidized to green ABTS^·+^ under oxidizing agents, exhibiting characteristic absorption peaks at 734 nm–405 nm. When antioxidants react with ABTS^·+^, the antioxidant donates a hydrogen atom or an electron, reducing the ABTS radical to a colorless state. First, ABTS solution (7 mM) and Na_2_S_2_O_8_ solution (2.45 mM) were prepared and mixed in a 1:1 volume ratio. The mixture was stirred overnight at 4 °C in the dark to generate stable ABTS^·⁺^. The mixture was diluted 50-fold with ethanol to obtain the ABTS^·+^ working solution. Next, 20 µL of PdM solutions at different concentrations (25, 50, 100 and 250 µg/mL) were added to a 96-well plate. For the blank group, 20 µL of deionized water was added to each well. Then 80 µL of the ABTS^·⁺^ working solution was added to each well and the plate was incubated in the dark at room temperature for 30 min. Finally, the absorbance of the samples was measured at 734 nm using a microplate reader. The scavenging capacity is determined by the reduction in absorbance. DPPH (2,2-diphenyl-1-picrylhydrazyl) is a commonly used for evaluating antioxidant activity. This test is based on the color change of DPPH radicals during the reaction. When antioxidants react with DPPH radicals, the DPPH transforms from a purple compound into a pale yellow or colorless compound. 20 µL of different concentrations of PdM (25, 20, 100 and 250 µg/mL) were added to a 96-well plate. For the blank group, 20 µL of deionized water were added to each well. Then, 100 µL of a 100 µM DPPH ethanol solution was added to each well. After incubating at room temperature in the dark for 30 min, the absorbance of the samples was measured at 517 nm using a microplate reader. DPPH scavenging rate of the sample was determined by the degree of absorbance reduction.

### In Vitro cytotoxicity assays

Cell counting kit-8 (CCK-8) and Live/Dead assays were utilized for evaluating the cytotoxicity as follows: For CCK-8 assay, HCECs were planted first in 96-well plates overnight at a density of 8 × 10^3^ cells per well. Different concentrations of PdM (5, 10, 25, 50, 100, 250 µg/mL) were added to the complete medium and incubated at 37 °C with 5% CO_2_ for 24 h. After incubation for 24 h, 100 µL of CCK-8 solution was added to each well according to the manufacturer’s protocol. The absorbances were then measured by a microplate reader (M5 SpectraMax, Thermo Fisher Scientific, USA) at 450 nm. The results were expressed as a percentage of the control. For Live/Dead assay, HCECs were planted first in 96-well plates overnight at a density of 5 × 10^3^ cells per well. Different concentrations of PdM (5, 10, 25, 50, 100, 250 µg/mL) were added to the complete medium and incubated at 37 °C with 5% CO_2_ for 24 h. After incubation for 24 h, the Calcein-AM and PI reagents were added to each well according to the instructions of the Calcein-AM/PI cell viability/cytotoxicity assay kit and incubated for 30 min at 37 °C in the dark. The fluorescent images were then photographed using an inverted fluorescence microscope (DMi8, Leica, GER).

### In vitro cellular uptake

To evaluate the cellular uptake capacity of HCECs for PdM, PdM was labeled with FITC. The capacity of HCECs to internalize PdM was evaluated using confocal microscopy and flow cytometry. For confocal microscopy imaging of PdM cellular uptake: First, HCECs were seeded onto round coverslips in a 96-well plate and cultured for 1 to 2 days. Cells were treated with medium containing 100 µg/mL FITC-PdM for 30 min, 1 h, 2 h, or 3 h, respectively, followed by three washes with PBS. Next, the cell membranes were stained with the far-red fluorescent probe (DiD, Biosharp, China) for 20 min, followed by three washes with PBS. Then, cells were fixed with 4% PFA for 30 min and stained with an anti-fluorescence quencher containing DAPI. A confocal laser scanning microscope (LSM 880, Zeiss, GER) was used to observe the distribution of PdM in cells. To assess the uptake capacity of HCECs for PdM by flow cytometry, HCECs were seeded into 6-well plates and cultured for 1–2 days. Cells were treated with medium containing 100 µg/mL FITC-PdM for 30 min, 1 h, 2 h, and 3 h, respectively. After three washes with PBS, the cells were digested with trypsin and centrifuged. Then, the cells were resuspended in PBS and the FITC fluorescence expression of the samples was measured using a flow cytometer (BD C6 Plus, BD, USA).

### In vitro anti-oxidative stress of PdM

To investigate the ability of PdM to scavenge intracellular ROS, CM-H2DCFDA and dihydroethidium (DHE) probes were used to validate HCECs stimulated by oxidative stress (H₂O₂). The NC (negative control) group was not exposed to H₂O₂, while the PC group was exposed to H₂O₂ but not incubated with PdM. Briefly, 8 × 10³ cells were seeded into 96-well plates and cultured overnight. The NC group received complete medium, the PC group received complete medium containing 200 µM H₂O₂, and the PdM group received complete medium containing various concentrations of PdM (0, 50, 100 and 250 µg/mL) along with 200 µM H₂O₂. After overnight incubation, 5 µM CM-H₂DCFDA or DHE working solution was added to each well, followed by continued incubation for 30 min. Fluorescent images of the HCECs were then captured using an inverted fluorescence microscope (DMi8, Leica, GER).

### Protection of F-actin proteins

HCECs were seeded at a density of 5 × 10⁴ cells per well in a 24-well plate, with round coverslips placed at the bottom. After culturing for 24 h, the NC group was replaced with fresh complete medium. And the modeling group was supplemented with complete medium containing 200 µM H₂O₂. PdM group was supplemented with complete medium containing different concentrations of PdM (25, 50, and 100 µg/mL), as well as 200 µM H₂O₂. After culturing for a further 24 h, the cells were washed three times with PBS, then fixed with 4% paraformaldehyde at room temperature for 15 min. The cells were then permeabilized with 0.5% Triton X-100 for five min and washed three times with PBS. Cytoskeletal proteins were stained with rhodamine-labeled phalloidin for 30 min. After mounting with an anti-fluorescence quenching mounting medium containing DAPI, cells were imaged using a laser confocal microscope (LSM 880, Zeiss, GER).

### Immunofluorescence Staining

HCECs were seeded at a density of 5 × 10⁴ cells per well in 24-well plates with round coverslips placed at the bottom. After 24 h of incubation at 37 °C with 5% CO₂, the NC group was replaced with fresh complete medium. The PC (positive control) group received complete medium supplemented with 200 µM H₂O₂. The PdM group received complete medium supplemented with PdM at different concentrations (25, 50, and 100 µg/mL) plus 200 µM H₂O₂. After 24 h further culture, cells were fixed with 4% paraformaldehyde at room temperature for 15 min and washed three times with PBS. Cells were blocked with 5% BSA at room temperature for 1 h. Primary antibodies (Claudin-1, Proteintech, 1:200) diluted in 5% BSA were added to corresponding wells. After incubating at room temperature for 3 h, the cells were washed three times with PBST. The fluorescently labelled secondary antibody, diluted in 5% BSA, was then added and the cells were left to incubate in the dark at room temperature for 2 h. Following three washes in PBST, cells were mounted with an anti-fluorescence quenching mounting medium containing DAPI and imaged using a laser confocal microscope (LSM 880, Zeiss, GER).

### DED model mice

The animal experiment was approved by the Laboratory Animal Ethics Committee of Eye Hospital, Wenzhou Medical University and conducted in accordance with the guidelines (Approval No. YSG25072501). Female C57BL/6J mice (body weight 18–22 g, age 6–8 weeks) were purchased from Zhejiang Vital River Laboratory Animal Technology Co., Ltd. and fed at the Wenzhou Medical University Laboratory Animal Center. The mice were housed in a well-ventilated area with humidity maintained at 30 ± 3% and a temperature of 23–25 °C and exposed to a regular light/dark cycle (12 h/12 h) and had free access to food and water. All of the experimental mice were confirmed to have no defects of the iris or cornea. An experimental animal model of DED was established using scopolamine hydrobromide (Scop). Briefly, Scop solution (0.5 mg/0.2 mL) was administered via intraperitoneal subcutaneous injection four times daily for seven consecutive days. The specific grouping was as follows: (1) NC group: normal mice without DED modeling, administered with saline eye drops; (2) PC group: DED mice treated with saline eye drops; (3) SH group: DED mice treated with commercial sodium hyaluronate eye drops (0.3% sodium hyaluronate, URSAPHARM, GER); (4) PdM group: DED mice treated with 100 µg/mL PdM solution eye drops. The eye drops were administered three times daily (5 µL per eye) for seven consecutive days.

### Therapeutic efficacy assessment

Clinical indicators for DED include fluorescein sodium staining scores and the phenol red cotton wool tear secretion test. The phenol red thread was cut to an appropriate length in advance. During the test, phenol red thread was placed at one-third of the palpebra inferior conjunctiva for 30 s, and the length of the impregnated portion was measured using the built-in ruler of the reagent kit. Corneal epithelial defects were assessed via corneal fluorescein staining under a slit-lamp microscope. The slit lamp with cobalt blue illumination was used to take corneal photographs and the clinical scoring criteria were used to score them. 3 µL of fluorescein sodium solution was instilled and observed under cobalt blue light using a slit-lamp microscope. Scores from 0 to 4 were assigned to each quadrant based on the following criteria: The cornea was divided into five sectors: superior, inferior, nasal, temporal, and central. Each sector received a grade of 0–3: 0) no staining; (1) mild punctate staining, < 30 spots; (2) punctate staining > 30 spots, but not diffuse; (3) diffuse staining but no positive plaques; (4) positive plaques. The final corneal fluorescein staining score was calculated by summing the scores from the five different areas.

### Histological evaluation

On day 7 of treatment, mice were euthanized, and eyeballs along with the entire conjunctiva were harvested for histological examination. The eyeballs were gently rinsed with physiological saline. The eyeballs then underwent gradient dehydration using sucrose solutions of varying concentrations. They were frozen in an optimal cutting temperature compound, sectioned into 10 μm-thick serial slices, and stored at −80 °C. The tissues were stained with haematoxylin and eosin (H&E) and periodic acid–Schiff (PAS) and photographed using an upright microscope (Axio Imager 2, Zeiss, Germany) to evaluate histological changes.

### Proteomics analysis of the cornea and conjunctiva

To further analyze the therapeutic efficacy of PdM in DED mice, corneal and conjunctival tissues from each group were obtained for proteomic analysis. On day 7 of treatment, the experimental mice were euthanized, and corneal and conjunctival tissues were harvested for proteomic analysis. Proteins with missing values exceeding 90% in all samples were excluded, and missing values were imputed using the k-nearest neighbors (kNN) method. Samples were analyzed using the Reactome database (https://reactome.org/).

### Immunofluorescence staining of corneal conjunctival tissue

The frozen sections were rewarmed to room temperature for 5 min and washed three times with TBST solution. Tissue sections were permeabilized with 0.3% Triton X-100 for 10 min, followed by blocking with 5% BSA at room temperature for 1 h. Primary antibodies (MUC5AC, IL-6, NF-κB, MMP-9, TNF-α, Proteintech, 1:250) diluted in 5% BSA were incubated with tissue at 4 °C overnight. After incubation, sections were washed three times with PBS for 5 min. Tissue sections were incubated with appropriately diluted, fluorophore-grafted secondary antibody at room temperature in the dark for 2 h. Subsequently, tissue sections were washed three times with TBST for 5 min each. After mounting with an anti-fluorescence quenching mounting medium containing DAPI, immunofluorescence images of the cornea and conjunctiva were captured and recorded using a laser confocal microscope (LSM 880, Zeiss, GER).

### Safety assessment of PdM

To investigate the long-term toxicity and biocompatibility of PdM, animal experiments were conducted to validate its safety. Eight mice were randomly divided into two groups: negative control (NC) group and PdM group. Mice in the NC and PdM groups received eye drops of saline solution and PdM (100 µg/mL), respectively, three times a day (5 µL per time) for 7 consecutive days. Safety assessment of PdM was evaluated through corneal fluorescein staining, body weight measurement, intraocular pressure (IOP), and histopathological evaluation of mice. On Days 0 and 7, the corneas of experimental mice were stained with fluorescein sodium under cobalt blue light using a slit-lamp microscope. Body weights and IOP of mice were recorded on Days 0 and 7. Finally, the mice were euthanized, and eyeballs along with the entire conjunctiva were removed for histological sectioning. Concurrently, major organs, namely the heart, liver, spleen, lung, and kidney, were swiftly removed and fixed in H&E solution for subsequent biocompatibility evaluation. The specific procedure was performed as described previously. The specific procedure was performed as described previously.

### Statistical analysis

The results were expressed as mean ± standard deviation (SD). All statistical analyses were performed using GraphPad Prism and Origin software. An independent-sample t-test was used to compare between two groups. One-way analysis of variance (ANOVA) followed by appropriate post hoc tests was used to compare means among multiple groups. Statistical significance was interpreted as follows: *p* > 0.05, not significant (ns); *p* < 0.05, significant (*); *p* < 0.01, highly significant (**); *p* < 0.005, very highly significant (***); and *p* < 0.001, extremely significant (****).

## Results and discussion

### Preparation and characterization of PdM

**Fig. 1:**
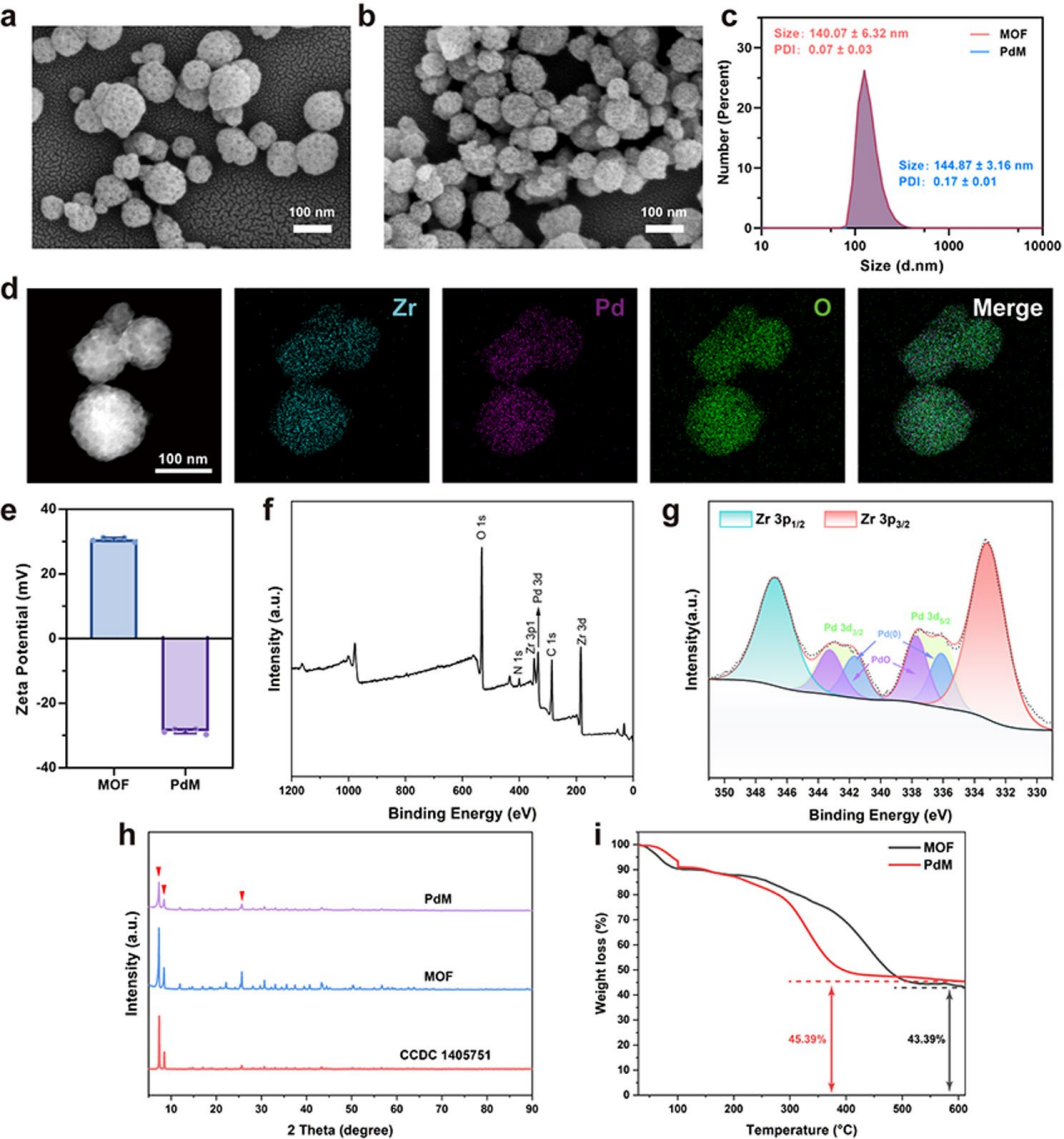
Characterization of MOF and PdM. (**a**, **b**) SEM images of MOF and PdM. (**c**) DLS and PDI results of MOF and PdM. (*n* ≥ 3) (**d**) TEM-EDS results for MOF and PdM. (**e**) Zeta potential of MOF and PdM. (*n* ≥ 3) (**f**) XPS spectrum of PdM. (**g**) XPS spectrum of Pd in PdM. (**h**) XRD patterns of MOF and PdM. (**i**) Thermogravimetric analysis curves of MOF and PdM. Data are means ± standard deviation (s.d.) (n $\:\ge\:$ 3)

PdM was synthesized on the ordered mesoporous MOF synthesized by the Hofmeister ion-mediated soft template method [38, 39]. The synthesis process of PdM was shown in Scheme 1. Pluronic^®^ F-127 was utilized as a template to generate continuous large mesopores within the microporous framework of UiO-66-NH₂ MOF. As a Hofmeister ion, Br^−^ was introduced into the system as a mediator to promote oriented crystallization. Through precise control of synthesis parameters, monodisperse spherical mesoporous MOFs with a particle size of approximately 120 nm were successfully obtained. The synthesis process of PdM is shown in Scheme 1. SEM images showed that the pristine MOF had a large number of uniformly distributed mesopores (Fig. 1a), and the PdM nanoparticles retained the porous structure after Pd loading (Fig. 1b), which provided a structural basis for ROS binding and scavenging. Dynamic light scattering (DLS) results showed that the average size of MOF and PdM increased from 140.07 ± 6.32 to 144.87 ± 3.16 nm, with PDI from 0.07 ± 0.03 to 0.17 ± 0.01 after loading Pd (Fig. 1c). Transmission electron microscopy (TEM) and energy-dispersive X-ray spectroscopy (EDS) results further confirmed the uniform distribution of mesoporous structures within the PdM surface and the distribution of Zr, Pd, and O elements (Fig. 1d). As shown in Fig. 1e, anchoring Pd nanoparticles caused the MOF’s ζ-potential to shift from positive to negative. X-ray photoelectron spectroscopy (XPS) spectrum results confirmed the presence of C, N, O, Zr, and Pd elements in PdM (Fig. 1f) and the presence of zero-valent Pd and PdO (Fig. 1g). The XPS spectrum of Pd 3 d exhibited two peaks at 336.8 and 342.4 eV, corresponding to Pd⁰ 3d⁵/² and Pd⁰ 3d³/², respectively. This indicates the coexistence of metallic and oxidized Pd states on PdM.

As shown in Fig. 1h, the synthesized MOF and PdM showed distinct characteristic peaks at 2θ = 7.3°, 8.44°, and 25.7°. These peaks closely matched the standard card for MOF (CCDC 1405751), indicating that the framework structure of MOF remained well-preserved after the modification process. The characteristic Pd peaks were not observable in the X-ray diffraction (XRD) pattern, probably due to the high dispersion and low concentration of Pd nanoparticles. Figure 1i displays the TGA curves of MOF and PdM in an air atmosphere across the temperature range of 100–600 °C. Due to the oxidative decomposition of C, N, H, and other elements, both MOF and PdM exhibited continuous weight loss between 100 and 500 °C. Within the 500–600 °C range, the samples’ weight tended to stabilize. The final residual mass percentages of MOF and PdM were 43.39% and 45.39%, respectively, attributable to the presence of oxidation products of Zr and Pd elements. Consequently, the calculated palladium content in PdM was determined to be 1.6%.

### Enzyme-like activity and ROS scavenging capacity of PdM

**Fig. 2:**
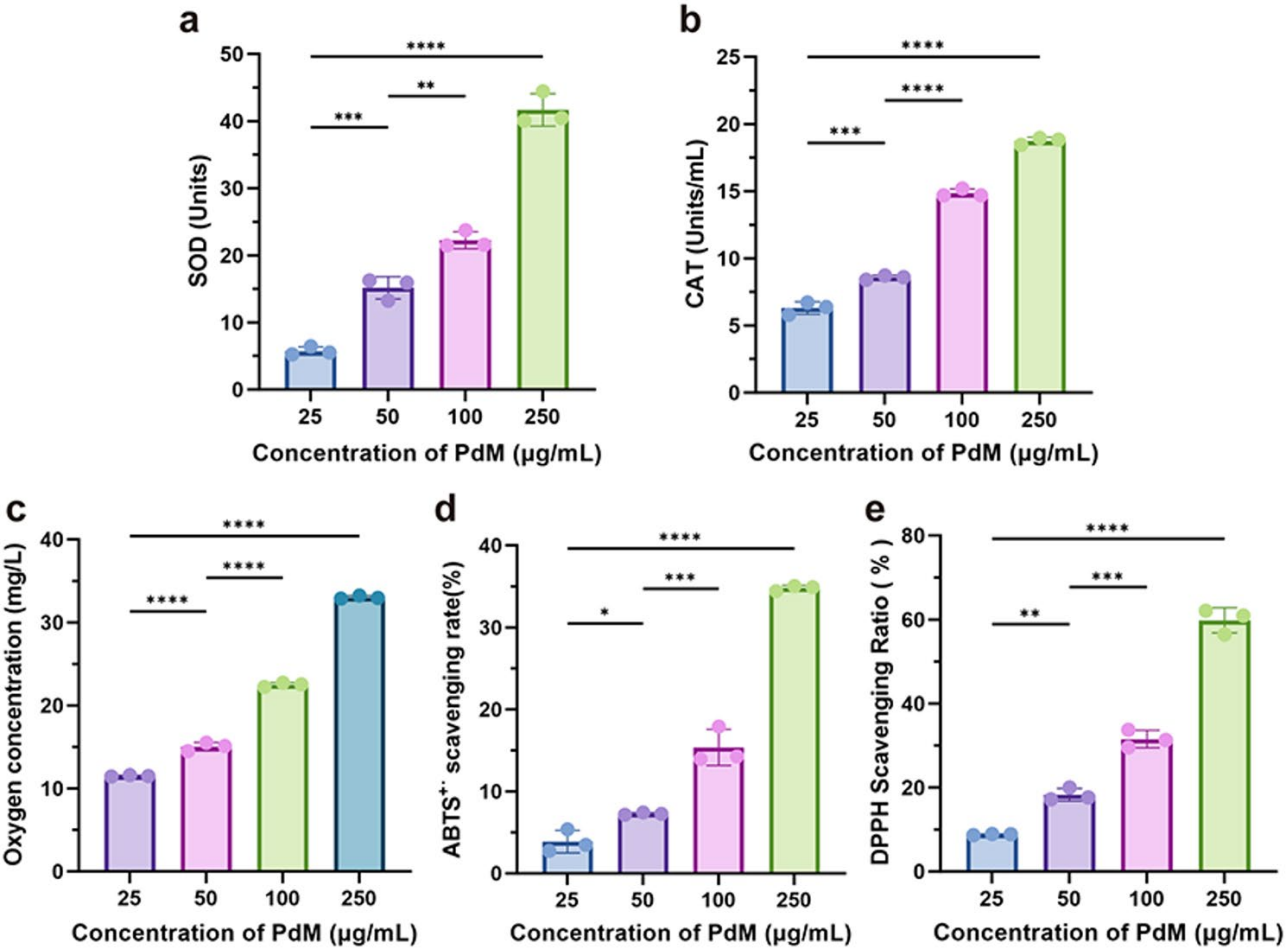
Enzyme-like activity and ROS scavenging capacity of PdM. (**a**, **b**) Detection of SOD and CAT mimicking activity of PdM at different concentrations. (SOD: superoxide dismutase; CAT: catalase) (*n* ≥ 3). (**c**) H_2_O_2_ scavenging capacity of PdM. (**d**, **e**) ABTS^·+^ and DPPH scavenging ability of PdM (*n* ≥ 3). Data are means ± s.d. (n $\:\ge\:$ 3). *p < 0.05, **p < 0.01, ***p < 0.005, **** *p* < 0.001, ns: not significant

Due to the presence of numerous ROS-active binding sites on its surface, Pd nanozymes can bind to hydrogen peroxide, singlet oxygen, hydroxyl radicals, and other ROS species. They reduce these ROS to H₂O through O–O bond breaking and successive hydrogen transfer steps. When subjected to excessive oxidative stress, Pd nanozymes can mimic the activities of SOD and CAT to scavenge excess ROS. SOD-mimetic activity and CAT-mimetic activity of PdM at different concentrations (25, 50, 100, and 250 µg/mL) were detected. As shown in Fig. 2a, the SOD activity of PdM at a low concentration (25 µg/mL) was approximately 5.53 ± 0.64 Units. When the concentration increased to 250 µg/mL, the SOD activity increased to 41.66 ± 2.42 Units. Significant differences in SOD activity were observed across different concentrations of PdM, indicating that the SOD-mimetic activity of PdM increased with concentration. As shown in Fig. 2b, CAT-mimetic activity of PdM at a concentration of 25 µg/mL was approximately 6.3 ± 0.46 Units/mL. As the concentration of PdM was raised to 250 µg/mL, CAT-mimetic activity increased to 18.74 ± 0.27 Units/mL. CAT-mimetic activity of PdM exhibited a concentration-dependent increase. This indicated that PdM exhibited both SOD-mimetic and CAT-mimetic activities, enabling it to scavenge excess ROS.

To further investigate the scavenging capacity of PdM toward ROS, we evaluated its scavenging ability for various free radicals. In the H₂O₂ scavenging experiment (Fig. 2c), the dissolved oxygen concentration in the solution increased with rising PdM concentration. This indicated that PdM possesses H₂O₂ scavenging capability, enabling it to rapidly eliminate H₂O₂ from the environment. Figure 2d-e demonstrated that PdM possessed the ability to scavenge ABTS^·+^ and DPPH, with its scavenging efficiency increasing with concentration. In summary, PdM exhibited multiple enzyme- mimetic activities and was able to efficiently scavenge various free radicals.

### In vitro biocompatibility of PdM

**Fig. 3:**
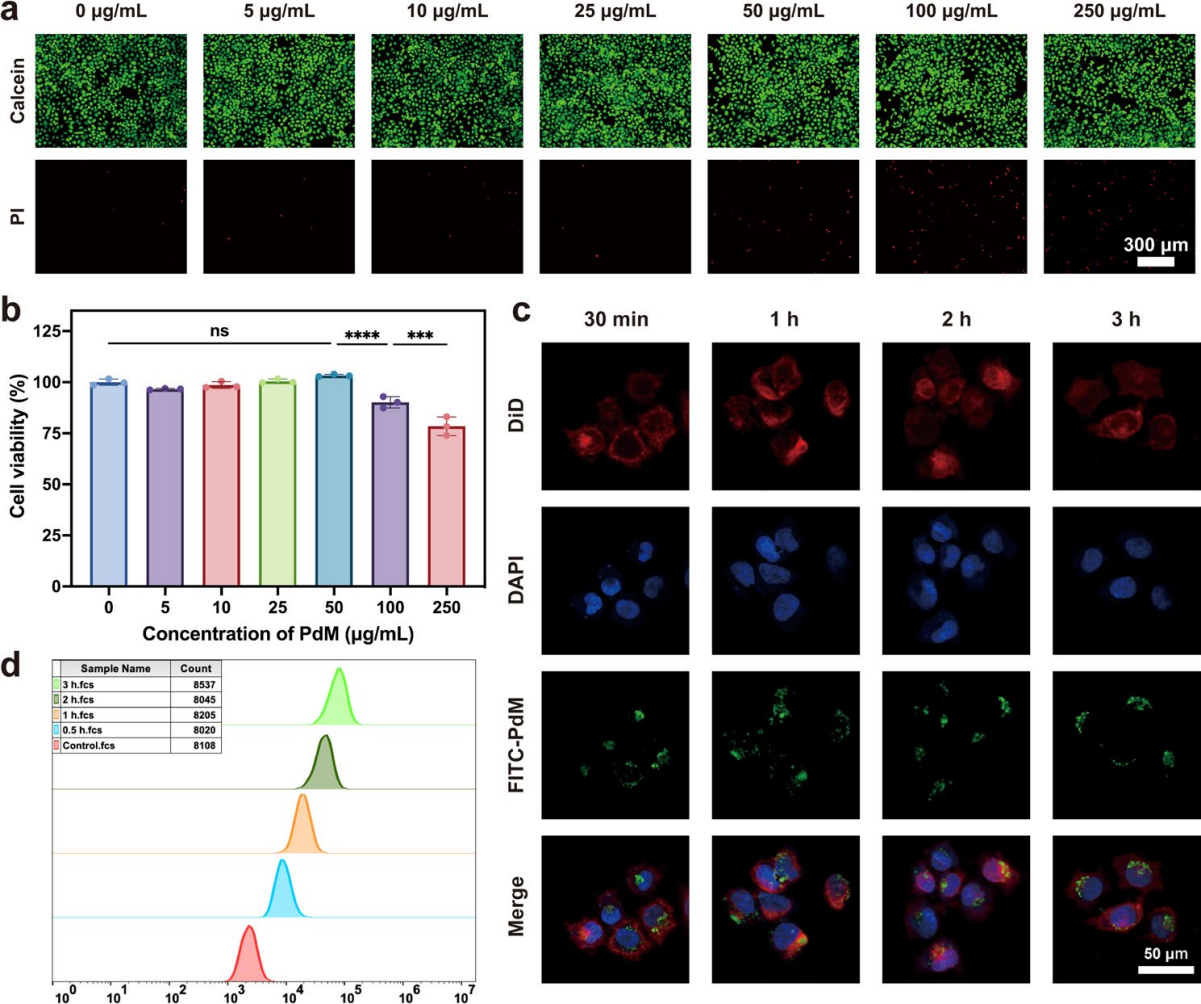
Biocompatibility and cell uptake evaluation of PdM in vitro. (**a**) Fluorescence image of the Calcein-AM/PI cell viability/cytotoxicity assay (*n* ≥ 3). Scale bar: 300 μm. (**b**) Cytotoxicity evaluation of different concentrations of PdM incubated with HCECs for 24 h (*n* ≥ 3). (**c**) Fluorescent images of HCECs internalizing PdM after different incubation times (*n* ≥ 3). Scale bar: 50 μm. (**d**) Flow cytometry results of PdM uptake by HCECs (*n* ≥ 3). Data are means ± s.d. (n $\:\ge\:$ 3). *p < 0.05, **p < 0.01, ***p < 0.005, **** *p* < 0.001, ns: not significant

When using PdM as an eye drop for DED treatment, its optimal concentration is critical for in vivo therapeutic efficacy. Human corneal epithelial cells (HCECs) were employed as a safety-validated cell line to investigate the optimal PdM concentration via live/dead staining and CCK-8 assays. As shown in Fig. 3a, HCECs exhibited no significant morphological changes at PdM concentrations below 100 µg/mL compared to untreated cells (0 µg/mL), with only a few dead cells observed. When the PdM concentration exceeded 100 µg/mL, red fluorescence increased in proportion to the concentration; however, no significant red fluorescence signal was detected. This suggests that PdM can be used safely at concentrations below 100 µg/mL. Consistent with the live/dead cell staining results, Fig. 3b indicated that HCECs exhibited no significant reduction in cell viability or cell death at PdM concentrations below 100 µg/mL. However, when PdM concentrations increased to 250 µg/mL, cell survival rates decreased to approximately 80%, with no significant difference between the two concentrations (*P* > 0.05). In summary, both the cytotoxicity assessment and the Calcein-AM/PI cell viability/cytotoxicity assay indicated that there was no apparent toxicity below 100 µg/mL. Consequently, this concentration may be considered the safe threshold for PdM application.

### Cellular uptake capacity of PdM

To investigate the uptake capacity of PdM by HCECs, FITC-PdM was employed in corresponding cellular experiments. Fluorescent images (Fig. 3c) revealed green fluorescence between the cell membrane and nucleus after 30 min of treatment, indicating that HCECs can internalize FITC-PdM into the cytoplasm. Flow cytometry results (Fig. 3d) showed an increase in fluorescence expression in the FITC channel over time. This suggests PdM accumulates within the cells. Consequently, PdM can be rapidly internalized by HCECs.

### In vitro anti-oxidative stress of PdM

**Fig. 4:**
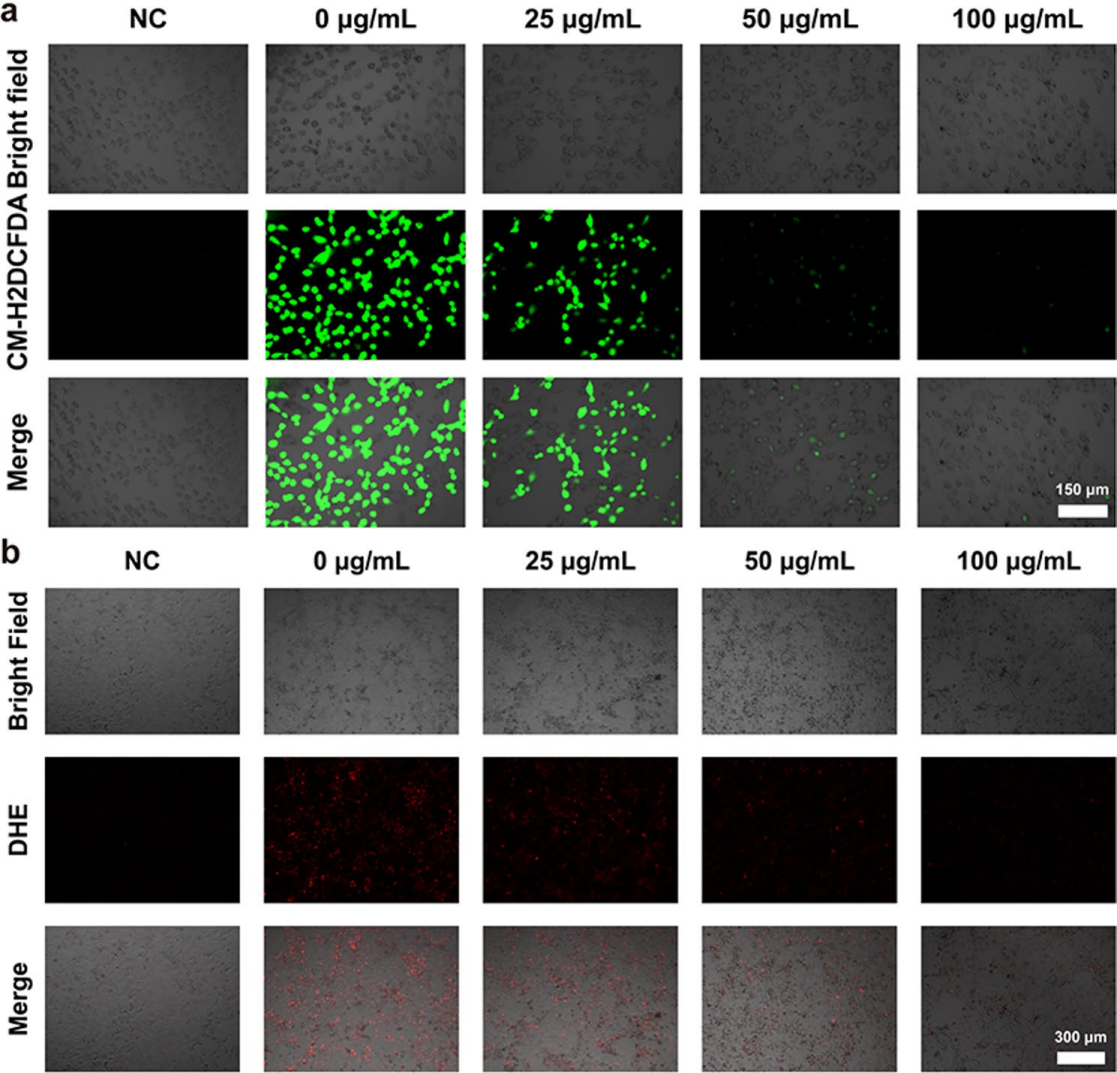
In vitro ROS scavenging and anti-inflammatory activity and protection against oxidative stress in HCECs of PdM. (**a**) Representative images of HCECs after different treatments using CM-H₂DCFDA staining (*n* ≥ 3). Scale bar: 150 μm. (**b**) Representative images of HCECs after different treatments using DHE staining (*n* ≥ 3). Scale bar: 300 μm

The vicious cycle of DED disease arises from factors such as environmental influences, light exposure, and elevated ROS levels in the tear film, leading to hypertonicity, apoptosis, inflammation, and reduced tear film stability. H₂O₂, a ROS, induces oxidative stress and apoptosis in HCECs, where disruption of the oxidation-antioxidation balance leads to cellular damage. To evaluate the antioxidant and ROS scavenging properties of PdM, H_2_O_2_ was used to construct a cellular model of oxidative stress in HCECs. CM-H₂DCFDA was employed to detect intracellular ROS, which is hydrolyzed by intracellular esterases into CM-H₂DCF — a compound unable to cross the cell membrane. Intracellular ROS oxidize the non-fluorescent CM-H2DCF to form fluorescent CM-DCF, enabling assessment of intracellular ROS levels. Following 200µM H₂O₂ stimulation of HCECs, the PC group exhibited increased green fluorescence (Fig. 4a), while PdM-treated groups showed significantly reduced fluorescence expression. Compared to 25 µg/mL, higher PdM concentrations (50 µg/mL, 100 µg/mL) resulted in markedly diminished fluorescence intensity. Dihydroethidium (DHE) is a widely used superoxide anion fluorescent probe that can be directly used for labeling live cells. After uptake by living cells, DHE undergoes dehydrogenation under the action of intracellular superoxide anions, producing a compound that binds to RNA or DNA to generate red fluorescence. This allows DHE to be used for detecting superoxide anion levels. Following 200µM H₂O₂ stimulation of HCECs, the PC group exhibited increased red fluorescence (Fig. 4b), whereas PdM-treated groups showed significantly reduced fluorescence expression. The red fluorescence intensity in PdM-treated groups decreased with increasing PdM concentration. Collectively, these results indicated that PdM effectively scavenged intracellular ROS levels in HCECs under oxidative stress stimulation.

### Protection of F-actin proteins

**Fig. 5:**
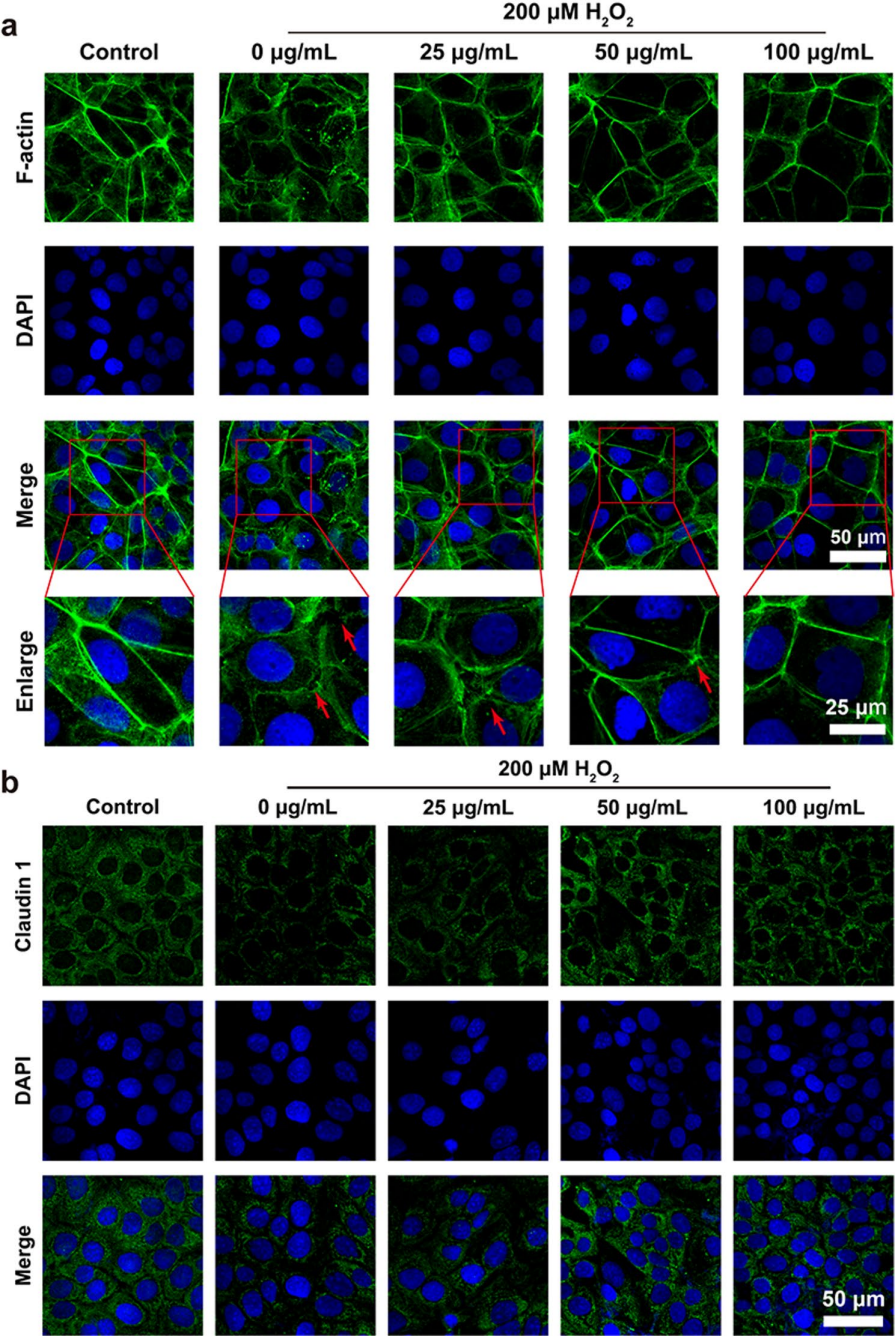
In vitro protection of cytoskeletal structures and tight junctions against oxidative stress by PdM. (**a**) Fluorescence images of HCECs stained with FITC-Phalloidin under different treatments (*n* ≥ 3). (**b**) Immunofluorescence staining images of Claudin 1 in 200 µM H_2_O_2_ stimulated HCECs after treatments (*n* ≥ 3). Scale bar: 60 μm

The cytoskeletal state and structural integrity of HCECs under oxidative stress were examined using co-staining methods, and the protective effect of PdM on cells under oxidative stress was evaluated. Fluorescently labeled phalloidin (marking cytoskeletal F-actin) and DAPI (marking cell nuclei) were used for co-staining to observe cellular structural integrity and morphological changes. Untreated HCECs served as the Control group. The H₂O₂ group was treated with 200 µM H₂O₂ to establish an oxidative stress-induced cellular damage model, evaluating cytoskeletal integrity under oxidative stress conditions. At the H₂O₂ treatment level, HCECs were additionally treated with varying concentrations of PdM to assess its protective effects. Results are shown in Fig. 5a. Compared to the Control group, the H₂O₂-treated group exhibited cytoskeletal collapse with markedly disrupted cell morphology and integrity, confirming successful induction of the oxidative stress model. At 25 µg/mL PdM treatment, actin structures remained largely intact with only slightly coarse local features. When the concentrations of PdM reached 50 µg/mL and 100 µg/mL, the cytoskeleton was normal and cellular structure was intact. This demonstrates that PdM possesses significant cytoprotective capacity, capable of counteracting H₂O₂-induced cytoskeletal structural damage and morphological changes.

### Protection of intercellular tight junctions

Hyperosmolar tears and oxidative stress in DED can impair the barrier function of HCECs by activating the p38/MAPK signaling pathway, leading to reduced expression of the tight junction protein Claudin-1. Immunofluorescence staining using Claudin-1 antibody (marking tight junction proteins) and DAPI was performed to examine the integrity of HCECs’ intercellular tight junctions under oxidative stress conditions, thereby evaluating PdM’s protective effect on these junctions. An oxidative stress-induced cellular damage model was established by treating HCECs with H₂O₂ to assess PdM’s impact on HCECs’ intercellular tight junctions under oxidative stress. As shown in Fig. 5b, the control group exhibited higher Claudin-1 expression, displaying intact cell contours and normal structural integrity. The H₂O₂-treated group showed significantly reduced Claudin-1 expression with markedly disrupted tight junctions. When PdM was co-administered with H₂O₂ treatment, green fluorescence expression increased in a concentration-dependent manner, demonstrating PdM’s protective effect on tight junctions. These findings highlighted the destructive impact of oxidative stress on cellular structural integrity, particularly its detrimental effects on tight junctions. The application of PdM effectively and reduced oxidative stress-induced damage and maintained the normal structure of tight junctions.

### In vivo therapeutic effects on DED

**Fig. 6:**
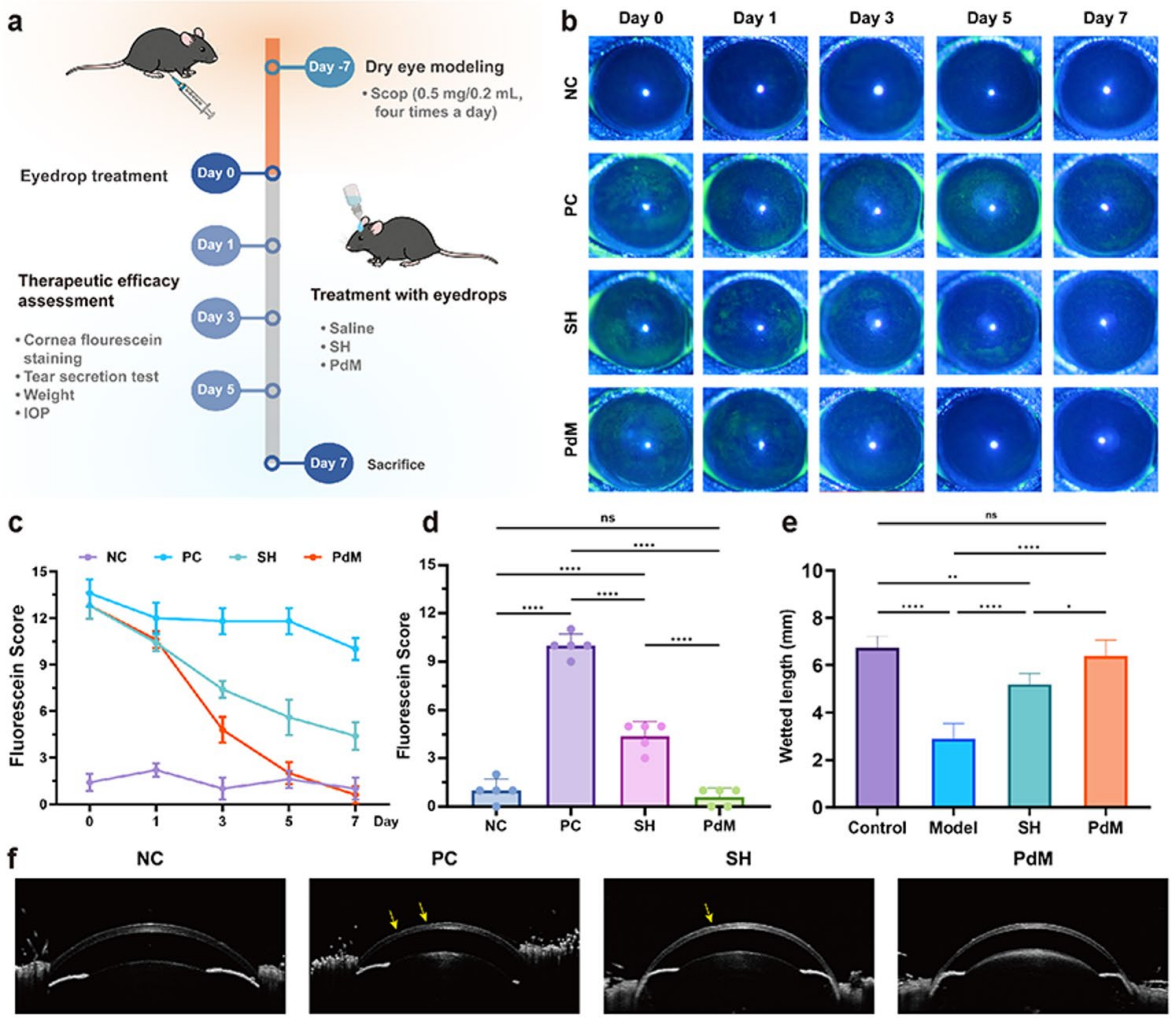
Evaluation of therapeutic effects on SCOP-induced DED mice. (**a**) Schematic diagram of the DED model establishment and treatment protocol. (**b**) Representative images of corneal fluorescein staining (*n* ≥ 3). (**c**) Results of corneal fluorescein staining scores (*n* ≥ 3). (**d**) Corneal fluorescein staining scores for each group on day 7 of treatment (*n* ≥ 3). (**e**) Results of phenol red tear secretion tests (*n* ≥ 3). (**f**) Representative OCT images of the anterior segment in mice from each group (*n* ≥ 3). Data are means ± s.d. (n $\:\ge\:$ 3). *p < 0.05, **p < 0.01, ***p < 0.005, **** *p* < 0.001, ns: not significant

The therapeutic efficacy of PdM for DED was evaluated using SCOP-induced DED mice (Fig. 6a). Corneal fluorescein staining was employed to assess corneal epithelial damage, which is an important diagnostic indicator for DED. After successful DED modeling, all mice underwent slit-lamp microscopy and corneal fluorescein staining at treatment days 0, 1, 3, 5, and 7. Tear secretion volume was measured on days 0 and 7. On day 0, the corneal epithelium in the untreated control (NC) group appeared smooth with no significant punctate staining (Fig. 6b). In contrast, the corneal epithelium in the other three groups exhibited opacity, showing distinct diffuse patchy and block-like staining, confirming successful establishment of the DED model. With prolonged treatment, corneal staining in the PdM group decreased significantly, with no obvious staining observed by Day 7. Consistent with the fluorescein staining scores shown in Fig. 6c, the PdM group’s corneal staining scores gradually decreased over the treatment period. On day 7 of treatment, the scores of PdM group showed no statistically significant difference compared to the control group (*p* > 0.05). Compared to the PC group, the SH group showed a significant decrease in scores (*p* < 0.001), while a significant difference remained between the SH and PdM groups (*p* < 0.001) (Fig. 6d). Experimental results demonstrated that PdM exhibited superior corneal epithelial cell protection effects compared to commercial sodium hyaluronate (SH) eye drops. Statistical analysis of tear secretion levels (Fig. 6e) revealed significantly reduced cotton thread wetting length (*p* < 0.001) and tear secretion volume in the PC group relative to the NC group, further confirming successful DED modeling. The SH group exhibited increased tear secretion compared to the PC group (*p* < 0.001), yet remained significantly different from the normal group (*p* < 0.05). In contrast, the PdM group restored tear secretion to normal levels, showing no statistical difference from the NC group, demonstrating PdM’s efficacy in DED treatment. Figure 6f showed OCT images of the anterior segment in mice from each group on day 7 of treatment. The PC group exhibited an irregular ocular surface with persistent corneal epithelial damage, while the SH group also showed defects, albeit milder than the PC group. Both the NC and PdM groups displayed smooth corneal morphology, indicating that the treatment of PdM promoted the recovery of corneal epithelial in DED mice.

### In vivo underlying mechanisms

To clarify the mechanism of PdM therapy for DED, proteomic analysis of corneal and conjunctival tissues was employed to investigate differential protein expression in tissues under different treatments. Mitogen-activated protein kinases (MAPKs) are one of the most important conserved protein kinase families in mammals, linking extracellular signals to intracellular mechanisms to regulate diverse cellular processes such as inflammation, immune responses, and apoptosis. Toll-like receptors (TLRs) are expressed on ocular surface cells in humans, and in the mouse, and recognize and respond to various microbes and endogenous stress ligands [40]. The inflammatory response in DED is believed to be triggered by hypertonicity and instability of the tear film, which correlates with the activation of MAPKs and TLRs, thereby inducing the production of various pro-inflammatory cytokines and matrix metalloproteinases (MMPs) on the ocular surface [41, 42].

**Fig. 7:**
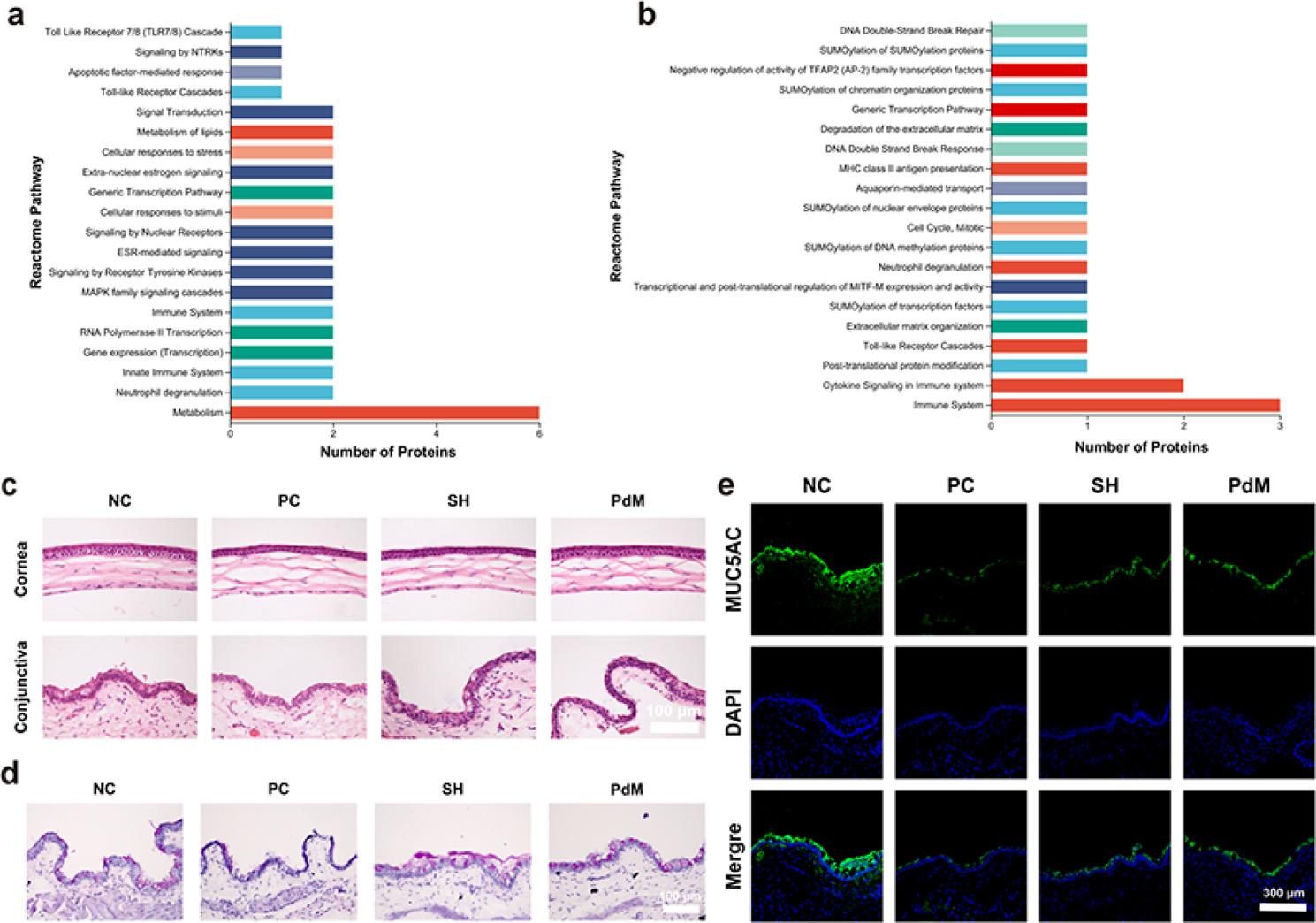
Proteomic and histopathological analysis, and immunofluorescence staining of of the anti-inflammatory effect of PdM on DED mice. (**a**) Reactome annotation analysis results for oxidative stress-related proteins up-regulated in the PC group relative to the NC group (n ≥ 3). (**b**) Reactome annotation analysis results for oxidative stress-related proteins downregulated in the PdM group relative to the PC group (n ≥ 3). (**c**) Representative HE-stained images of the cornea and conjunctiva from each group of mice (n ≥ 3). (**d**) Periodic acid-Schiff (PAS) stained conjunctival images from each group of mice (n ≥ 3). (**e**) Representative immunofluorescence images of MUC5AC in the conjunctiva of mice from each group (n ≥ 3). Data are means ± s.d. (n ≥ 3)

Based on the proteomics results, we explored the effects of PdM treatment on the expression of oxidative stress-related proteins. The Reactome database was used to analyze the expression of differentially expressed proteins across groups. As shown in Fig. 7a, the PC group exhibited protein-level upregulation in Metabolism, Immune System, MAPK signaling pathways, and Toll-like receptor proteins compared to the NC group. This reveals the potential activation of MAPKs and TLRs pathways in the corneal conjunctival tissues of mice with DED, leading to inflammatory expression. These pathways are closely linked to numerous others, including the NF-κB pathway, which has been shown to produce chemokines and pro-inflammatory cytokines. This also confirms with the feasibility of targeting the TLRs pathway for the treatment of DED in our team’s previous studies [29]. The results showed that the expression of proteins related to the immune system and Toll-like receptor cascade response in the corneal conjunctival tissues of mice were down-regulated in the PdM group compared with the NC group (Fig. 7b). This suggested that PdM may alleviate the progression of DED by inhibiting the activation of MAPKs and TLRs related signaling pathways in ocular surface tissues.

**Fig. 8:**
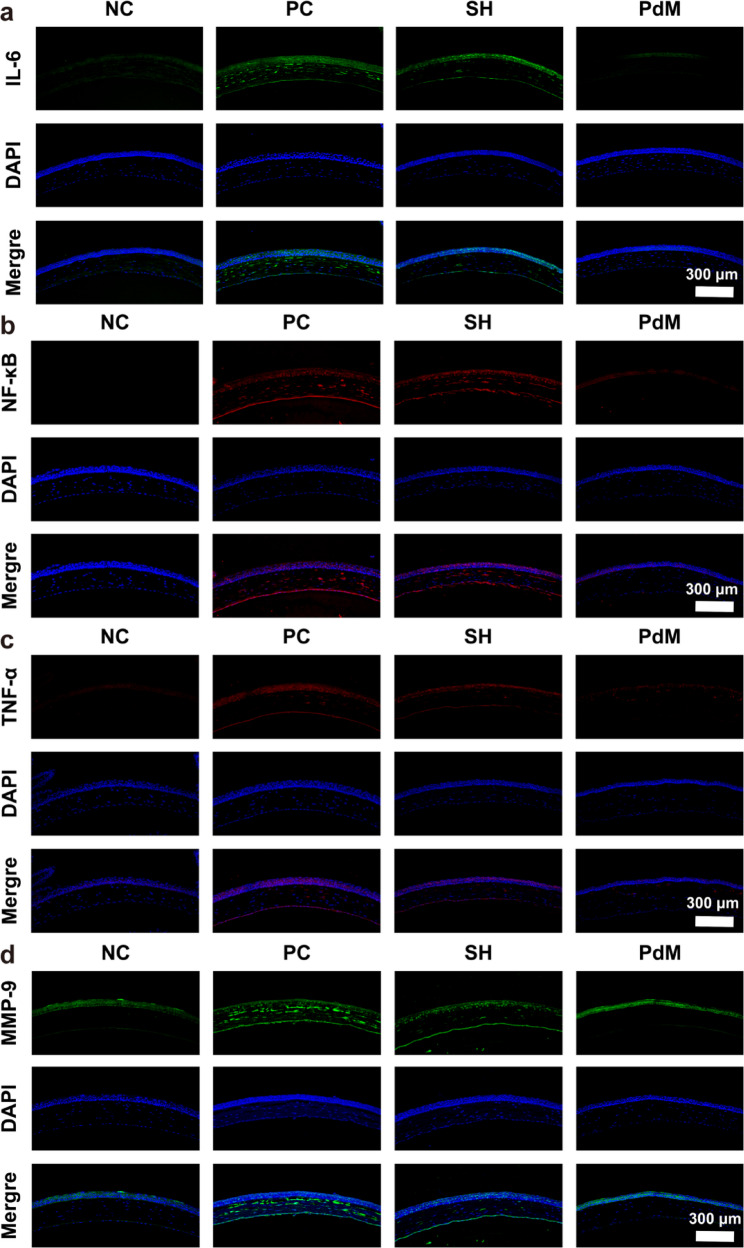
Fluorescent images of the cornea depict: (**a**) IL-6 (green), (**b**) NF-κB (red), (**c**) TNF- (red), and (**d**) MMP-9 (green), with cell nuclei stained by DAPI (blue) (*n* ≥ 3), scale bar: 300 μm

The therapeutic efficacy of PdM for DED was further evaluated using histopathological methods. Figure 7c-d showed the hematoxylin and eosin (H&E) staining results of corneal and conjunctival tissues, and the periodic acid-silver (PAS) staining results of conjunctival tissues, respectively. In the NC group, the corneal epithelium exhibited intact structure with tightly arranged cells, while the conjunctival tissue maintained complete architecture with orderly distribution of goblet cells. In contrast, the PC group exhibited disorganized corneal structure, significantly reduced corneal epithelial thickness, and atrophy of conjunctival goblet epithelial cells. The SH group showed some disorganization in corneal tissue arrangement and reduced corneal epithelial thickness. The PdM group demonstrated well-organized cellular arrangement and structural morphology in the corneal epithelium, with neatly arranged goblet epithelial cells in the conjunctival tissue. Immunofluorescence was employed to detect mucin 5AC (MUC5AC) secretion in the conjunctiva. The results (Fig. 7e) showed that by day 7, MUC5AC showed intense expression in the conjunctival tissue of the NC group. Expression of MUC5AC was markedly downregulated in both the PC and SH groups, while the PdM group showed increased expression compared to the PC and SH groups. This suggests that the expression of Mucin 5AC is reduced in the conjunctival tissues of mice in the PC group and SH group, and SH treatment may fail to protect conjunctival cupular epithelial cells. Immunofluorescence staining of frozen corneal sections from mice was performed to assess the expression of DED-related proteins. As shown in Fig. 8a-d, no expression of IL-6, NF-κB, TNF-α, or MMP-9 was detected in the corneal tissue of the NC group. In contrast, the PC group exhibited distinct fluorescent signals, indicating high expression of inflammatory-related factors in the corneal tissue of DED mice. The SH group also exhibited relatively strong fluorescence signals, indicating that commercial sodium hyaluronate artificial tears primarily function as ocular surface lubricants and cannot fundamentally alleviate ocular surface inflammation in DED mice. Comparatively, PdM group showed very weak fluorescence signals and low expression of inflammatory factors in corneal tissue, suggesting that PdM was able to reduce ocular surface inflammation.

It is worth noting that the significant therapeutic advantage of PdM over commercially available sodium hyaluronate (SH) eye drops is fundamentally due to their different mechanisms of action. SH is a traditional artificial tear that mainly temporarily alleviates DED symptoms through physical lubrication and moisturising effects [43–45]. However, it lacks the ability to directly intervene in the core pathological processes of DED: oxidative stress and the inflammatory response. Previous studies have shown that, while SH eye drops can improve tear film stability and subjective symptom scores, they have limited effects on key indicators such as promoting corneal epithelial repair, inhibiting inflammatory reactions and restoring tear secretion [46, 47]. Furthermore, SH can only temporarily reduce tear osmolarity through dilution and cannot eliminate the excessive ROS that perpetuate the DED cycle.

In contrast, PdM is a multifunctional nanozyme that simulates the multi-enzyme cascade activity of natural antioxidant enzymes. Specifically, PdM exhibits both superoxide dismutase (SOD) and catalase (CAT) like activity, decomposing ROS into water and oxygen and forming a complete ROS scavenging cascade reaction. This multi-enzyme synergistic effect enables PdM to effectively clear various ROS, including superoxide anions, hydrogen peroxide and hydroxyl radicals. It also inhibits the activation of MAPK and TLRs signaling pathways by scavenging intracellular ROS, thereby reducing the expression of NF-κB and the secretion of pro-inflammatory cytokines (IL-6, TNF-α) and matrix metalloproteinases (MMP-9), which breaks the oxidative stress-inflammation vicious cycle of DED. Therefore, not only can PdM rapidly alleviate symptoms, it can also repair the corneal epithelium, restore goblet cell density, promote tear secretion and achieve long-lasting therapeutic effects by targeting the basic pathological mechanisms of DED: oxidative stress and inflammation. The superior therapeutic effect of PdM compared to traditional SH eye drops is based on the fact that it has multiple enzyme activities rather than a single physical moisturising mechanism.

### Safety evaluation

**Fig. 9:**
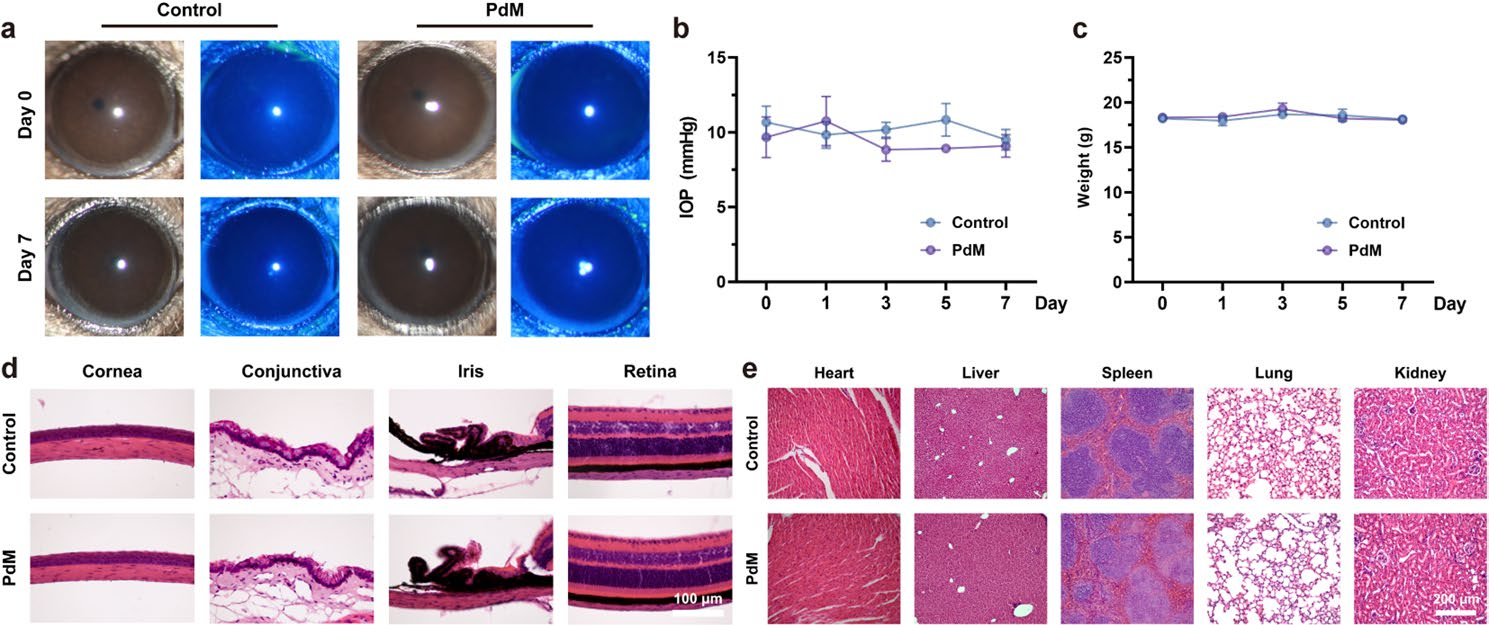
In vivo safety assessment of PdM. (**a**) Bright field and corneal fluorescein staining images before and after PdM instillation compared to PBS instillation (control group) (*n* ≥ 3). (**b**, **c**) Changes in body weight (**b**) and intraocular pressure (IOP) (c) before and after ocular administration of each group (*n* ≥ 3). (**d**) Representative HE stained images of cornea, conjunctiva, iris, and retina from each group of mice. (*n* ≥ 3) (**e**) Histopathological analysis of HE-stained tissues of heart, liver, spleen, lung, and kidney of mice in each group (*n* ≥ 3). Data are means ± s.d. (*n* ≥ 3). *p < 0.05, **p < 0.01, ***p < 0.005, **** *p* < 0.001, ns: not significant

To validate the in vivo biosafety and ocular compatibility of PdM, clinical examinations were conducted following drug instillation. Slit-lamp microscopy revealed no significant abnormalities in the anterior segment of mouse eyes, with clear corneas and no corneal epithelial damage (Fig. 9a). Throughout the experiment, intraocular pressure and body weight remained stable in mice (Fig. 9b, c). Hematoxylin and eosin (H&E) staining of paraffin-embedded ocular tissue sections revealed normal structural integrity in both the NC and PdM groups, with no significant pathological alterations observed in corneal, conjunctival, iris, or retinal tissues (Fig. 9d). HE staining of paraffin sections from major organ tissues revealed no significant pathological alterations in the PdM group compared to the NC group (Fig. 9e). These findings demonstrate the excellent compatibility and safety of PdM in ocular applications.

## Conclusion

DED is a serious eye disease whose global incidence is on the rise. However, conventional treatments are ineffective. The excellent therapeutic efficacy of PdM is attributed to its ability to target the core pathological process of DED: scavenging excess ROS to alleviate oxidative stress, which further inhibits the downstream inflammatory response and breaks the oxidative stress-inflammation vicious cycle. We designed a palladium-loaded MOF nanoparticle (PdM) with multiple antioxidant enzyme activities. PdM nanozyme can mimic antioxidant enzyme activities and exhibit multiple ROS scavenging abilities, thereby alleviating oxidative stress, restoring oxidation-antioxidant balance, and ultimately breaking the vicious cycle of DED. The results showed that HCECs undergo oxidative stress injury after H_2_O_2_ stimulation, resulting in elevated intracellular ROS levels accompanied by cytoskeletal collapse and disruption of tight junctions. PdM nanozyme treatment significantly improved these pathological manifestations. In DED mice, treatment with PdM nanozyme demonstrated excellent efficacy with significant improvements in clinical indicators: reduction in corneal damage, decrease in goblet cell injury, and reductions in inflammatory cytokine expression. Through seven days of topical administration to the ocular surface, the in vivo safety of PdM as an eye drop has been demonstrated. In summary, PdM nanozyme provides an effective solution for corneal damage repair in DED by scavenging excess ROS through multiple mimetic enzyme activities, thereby reducing ocular surface inflammation and tissue damage. This work provides a new design strategy for metal-loaded MOF nanozymes as ocular antioxidant therapeutics, and PdM eye drops hold great potential for DED treatment and other ocular surface diseases associated with oxidative stress.

## Data Availability

No datasets were generated or analysed during the current study.
